# Assessment of Cervical Cancer Prevention and Treatment Infrastructure in Belize

**DOI:** 10.1200/GO.21.00138

**Published:** 2021-08-05

**Authors:** Shane S. Neibart, Tiffany A. Smith, Jennifer H. Fang, Taylor Anderson, Abha Kulkarni, Jennifer Tsui, Shawna V. Hudson, Gregory L. Peck, Joseph S. Hanna, Natalia Largaespada Beer, Mark H. Einstein

**Affiliations:** ^1^ Rutgers Robert Wood Johnson Medical School, New Brunswick, NJ; ^2^ University of Queensland Ochsner Clinical School, Brisbane, QLD, Australia; ^3^ University of Southern California, Keck School of Medicine, Los Angeles, CA; ^4^ Rutgers Cancer Institute of New Jersey, New Brunswick, NJ; ^5^ Rutgers School of Public Health, Piscataway, NJ; ^6^ Ministry of Health, Belmopan, Belize; ^7^ Rutgers New Jersey Medical School, Newark, NJ

## Abstract

**PURPOSE:**

Belize has one of the highest cervical cancer burdens among Latin American and Caribbean countries, despite the implementation of national policies to increase access to prevention and treatment services. This study evaluates the policies, infrastructure, and workforce of the cervical cancer management system in Belize to inform capacity building efforts.

**METHODS:**

In 2018, health facility assessments were conducted across all six districts of Belize at the national pathology facility and 12 public facilities identified as critical to cervical cancer control. Human and infrastructure resource availability and existing policies related to cervical cancer screening and treatment services were assessed through a structured instrument.

**RESULTS:**

The public cervical cancer screening workforce in Belize consists of 75 primary care nurses and physicians—one per 1,076 screening-eligible women, with 44% conducting rural outreach. All districts have at least one screening facility, but 50% perform screening services only once per week. Colposcopy and loop electrical excision procedures are available in three and four districts, respectively; radical hysterectomy and chemotherapy are available in two districts; and radiation therapy is unavailable. Of essential pathology equipment, 38.5% were present and functional, 23% were present but nonfunctional, and 38.5% were unavailable. Additionally, 35% of supplies were unavailable at the time of assessment, and 75% were unavailable at least once in the 12 months before assessment.

**CONCLUSION:**

Public-sector cervical cancer management services differ among districts of Belize, with tertiary service availability concentrated in the largest district. Screening, outreach, and pathology are limited mostly by resource availability. This study characterizes the current capacity of services in Belize and pinpoints health system components for future investment and capacity-building efforts.

## INTRODUCTION

Cervical cancer is a preventable disease that disproportionately affects women in low- and middle-income countries, where nearly 87% of global cervical cancer deaths occur.^1^ In high-income countries, significant reductions in cervical cancer burden have been achieved through comprehensive preventative efforts including primary prevention with human papillomavirus (HPV) vaccination, secondary prevention with screening, treatment of precancerous lesions, as well as access to advanced treatment modalities such as radiation therapy.^2-5^ Improvements in such health system infrastructure with a focus on prevention has been shown to greatly reduce morbidity and mortality from cervical cancer.^6,7^

**CONTEXT**

**Key Objective**
What is the current capacity of cervical cancer prevention and treatment infrastructure in Belize?
**Knowledge Generated**
Cervical cancer prevention resources in Belize are strained by limited human resources and pathology infrastructure to support sustainable population-based screening efforts. Cervical cancer diagnostic and treatment resources are concentrated in the largest districts of Belize, creating geographic barriers to accessing critical interventions.
**Relevance**
Beyond characterizing the cervical cancer management system in Belize, this study provides a framework for evaluating  cervical cancer services in other low- and middle-income countries to support World Health Organization's targets for  accelerating the elimination of cervical cancer.

The high cervical cancer incidence and mortality rates in Latin American and Caribbean (LAC) countries exemplify the global inequity of cervical cancer and its association with poverty.^2,3^ In response, Nicaragua, Mexico, Argentina, El Salvador, Guatemala, and Honduras each began the implementation of national HPV vaccination and HPV-based or visual inspection with acetic acid (VIA)-based screening programs by 2017 as recommended by the WHO.^8^ Belize, a culturally and ecologically diverse LAC country of just under 400,000 citizens on the eastern coast of Central America, experiences some of the highest cervical cancer incidence and mortality rates among its peers. The 2018 estimates of cervical cancer incidence and mortality from the International Agency for Research on Cancer: Global Cancer Observatory found Belize to have age-standardized incidence and mortality rates of 28.0 and 16.2 cases per 100,000 population, respectively—far greater than those in other LAC countries (14.6 and 7.1 cases per 100,000 population, respectively) and North America (6.4 and 1.9 cases per 100,000 population, respectively).^9,10^

Recent policies implemented by the Ministry of Health (MOH) in Belize have aimed to increase the availability of HPV vaccination, cervical cancer screening services, and treatment options. Belize has been capitalizing on the opportunity to reduce cervical cancer incidence and mortality through implementations that have been performed and validated in other resource-constrained LAC nations. However, a detailed understanding of the Belizean health system must be considered before implementing newly targeted, fiscally sound, national health programs to maximize efficacy, value, and sustainability. The purpose of this cross-sectional study is to provide a baseline assessment of resource availability in Belize and explore areas of potential investment.

## METHODS

### Study Design and Sampling

This cross-sectional needs assessment was conducted in Belize in June 2018. Across all six districts of Belize, all public regional health facilities, the national health facility, the single national pathology facility, and any public health facilities that offered screening with VIA were included. VIA-based screening was used as an inclusion criterion for public health facilities because it serves as the current national cervical cancer screening approach. MOH officials who implemented the VIA program in Belize were consulted to ensure that all facilities offering VIA screening were included in the analysis. This study was approved by the Institutional Review Board at the Rutgers New Jersey Medical School and by the Director of Health Services at the MOH in Belize.

### Data Collection

The lead investigator (S.S.N.) underwent training with a MOH official (N.L.B.) and senior clinical investigator (M.H.E.) before beginning the study and performing the environmental scan of cervical cancer prevention in Belize. Cervical cancer prevention and treatment infrastructure and resources availability were assessed by conducting health facility assessments (HFAs) and one pathology facility assessment at the single national pathology facility in Belize. The Epidemiology Unit of the MOH and the Statistical Institute of Belize provided population-level demographic data for each district.

HFAs were performed using questionnaires constructed from international guidelines and subsequent incorporation of indicators from relevant literature in similar environments of care.^11,12^ Because Belize's official language is English, all questionnaires were conducted in English. Data fields within HFAs are described in Table 1. This cumulative information provided measures of cervical cancer screening and treatment capacity at each surveyed facility.

**TABLE 1:**
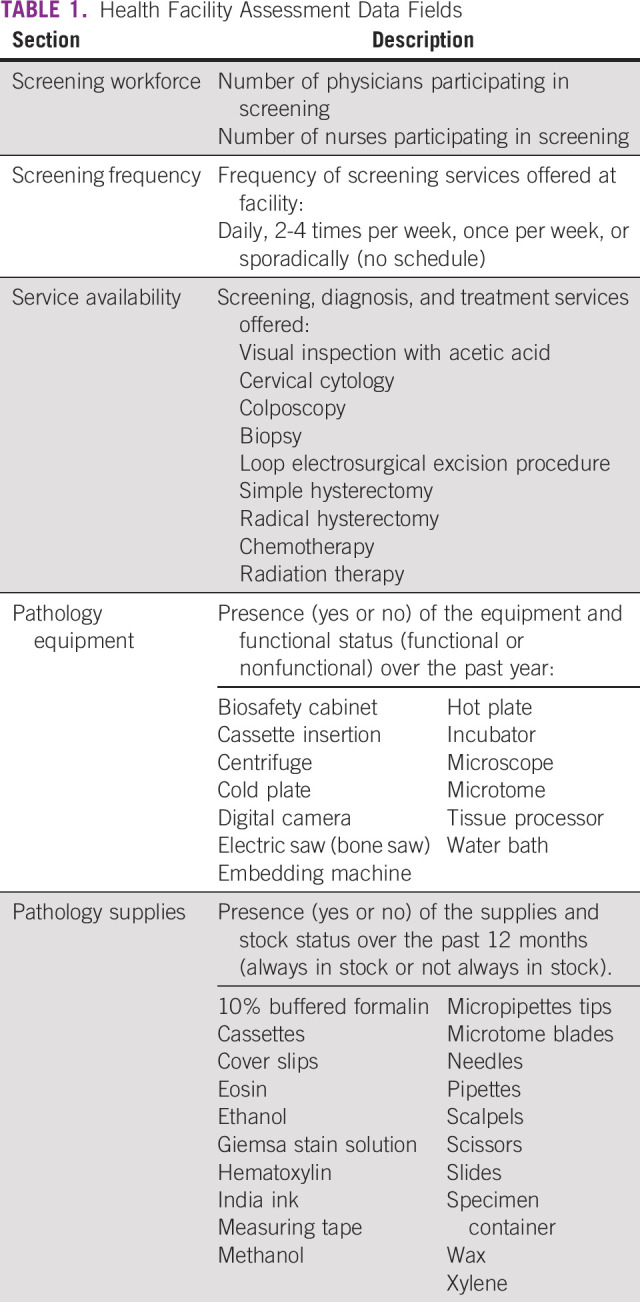
Health Facility Assessment Data Fields

The capacity of the single national pathology facility in Belize was evaluated by taking stock of commonly used materials (eg, hematoxylin), documenting supply shortages in the previous year, and documenting the functional status of all necessary equipment (eg, microtomes).

All HFA and pathology facility assessment data were collected using a secure Research Electronic Data Capture database and deidentified to maintain the privacy of each institution.

### Data Analysis

Population-level data from the MOH and the Statistical Institute of Belize were compiled by district (ie, Corozal, Orange Walk, Belize, Cayo, Stann Creek, and Toledo) and geographic region (Northern, Central, Western, and Southern). Facility assessment data were then categorized by district and region to estimate capacity for each area.

First, the screening and outreach workforces were estimated by totaling the number of nurses and general practitioners offering screening at each facility and those participating in regular outreach to rural health care centers or outposts. Regular outreach was defined as at least once per month. These data were tabulated for each district and normalized by the national population of women ages 25-70 years (screening-eligible women) in 2018.

The availability of services including cervical cytology and VIA was subsequently described by categorically recording the frequency with which the facilities offered these services: 1 day per week, 2-4 days per week, every day in a given week, or sporadically. The geographic distribution of offered preventative and treatment services were estimated by assessing the number of health facilities in each district that offered the following: cervical cytology, VIA with cryotherapy (single-visit approach), loop electrosurgical excision procedure, colposcopy, biopsy, pathology in house, simple hysterectomy, radical hysterectomy, chemotherapy, and radiation therapy.

Pathology equipment and supply availability was determined by direct investigation into essential equipment's presence on site, after which facility personnel responded to a binary question assessing equipment functionality. For each piece of essential equipment, the equipment was marked as present and functional, present but nonfunctional, or not present. The availability of 20 essential supplies for comprehensive cervical cancer screening including cytology and histology was determined by direct inquiry of supplies on site. Supplies were marked as either available or unavailable. Facility personnel also reported whether each item had been unavailable at any point during the 12 months before the study (June 2017-June 2018).

## RESULTS

### Facility Characteristics

A total of 13 facilities were assessed, including three regional health care centers, the national referral hospital, the national pathology facility, and eight community health centers that met the inclusion criteria of participating in cervical cancer prevention with VIA. Characteristics of the visited facilities are summarized in Table 2. Notably, the plurality of these facilities was located in Belize district, the most populous district of Belize, in contrast to the Toledo district, where only one facility was sampled.

**TABLE 2:**
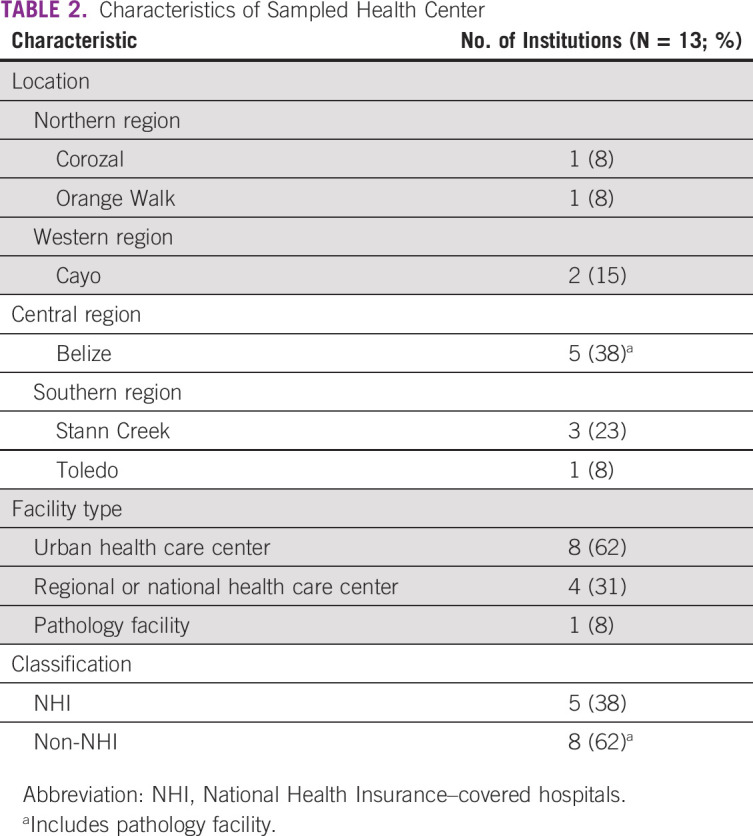
Characteristics of Sampled Health Center

### Workforce

**FIG 1:**
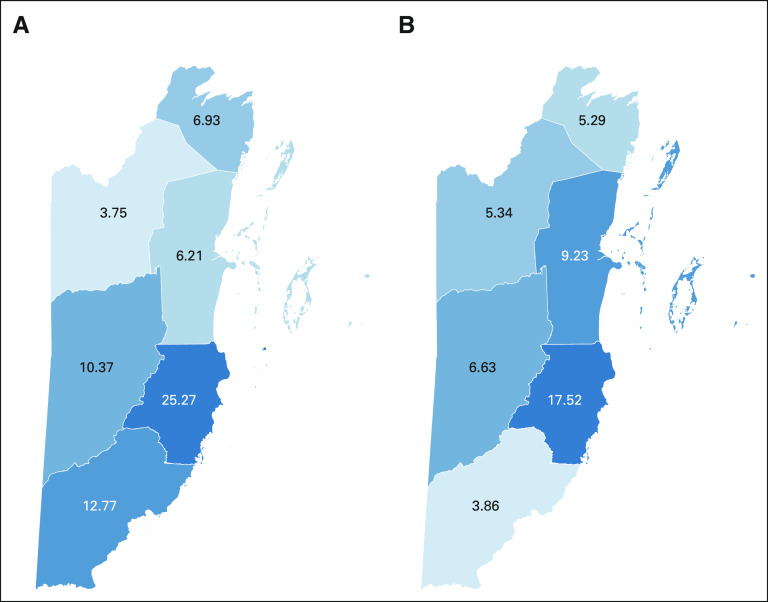
(A) Screening workforce per 10,000 screening-eligible women. (B) Outreach workforce per 10,000 screening-eligible women. Districts in clockwise order starting at the northernmost district: Corozal, Belize, Stann Creek, Toledo, Cayo, and Orange Walk.

In 2018, the cervical cancer screening workforce in Belize consisted of 75 nurses and physicians, with a density of approximately one health care provider for every 1,076 screening-eligible women. The workforce dedicated to outreach to rural facilities and communities consisted of 33 nurses and physicians, with a density of one health care provider for every 1,291 screening-eligible women living in rural communities. Stann Creek district had highest screening and outreach workforce density (25.27 and 17.52 personnel per 10,000 screening-eligible women, respectively), Orange Walk district had the lowest screening workforce density (3.75 personnel per 10,000 screening-eligible women), and Toledo had the lowest outreach workforce density (3.86 personnel per 10,000 screening-eligible women; Fig 1).

### Screening Services

**FIG 2:**
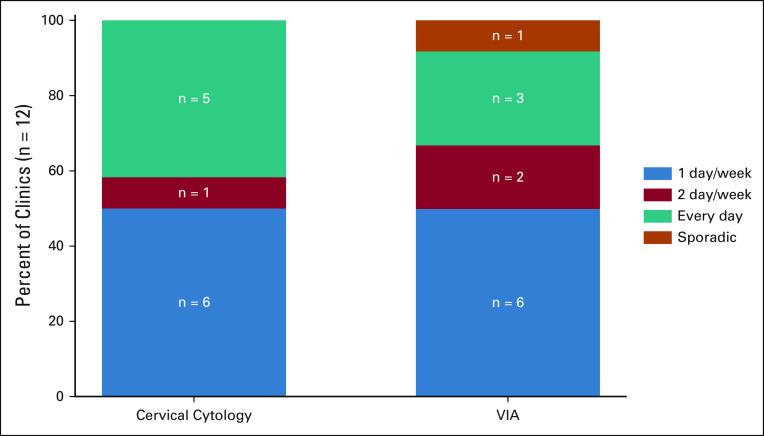
Screening service availability. VIA, visual inspection with acetic acid.

**FIG 3:**
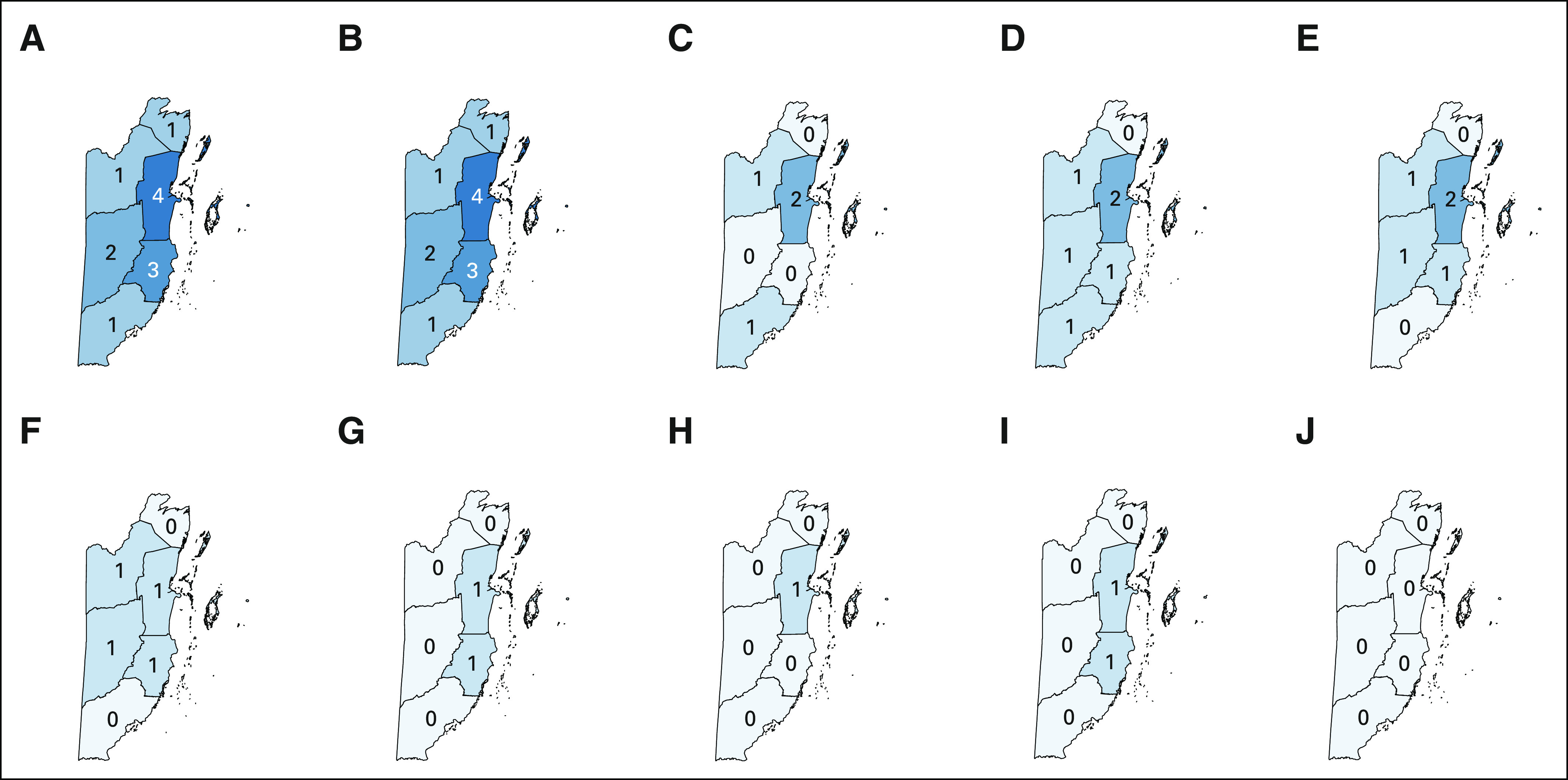
Number of facilities offering select services. (A) Cervical cytology. (B) VIA. (C) Colposcopy. (D) Biopsy. (E) LEEP. (F) Simple hysterectomy. (G) Radical hysterectomy. (H) Pathology in house. (I) Chemotherapy. (J) Radiation therapy. Districts in clockwise order starting at the northernmost district: Corozal, Belize, Stann Creek, Toledo, Cayo, and Orange Walk. Note: Chemotherapy was only offered in one facility sampled (located in Belize District), but an additional facility was identified as affiliated with the public health care system in Stann Creek. LEEP, loop electrosurgical excision procedure; VIA, visual inspection with acetic acid.

Screening service availability of 12 facilities that offered cervical cytology and VIA was described in Figure 2. Notably, 50% of facilities performed VIA (n = 6) only 1 day a week, and 50% of facilities performed cervical cytology (n = 6) only 1 day per week (Fig 2). Nearly half of all facilities (n = 5) provided cervical cytology every day, and only 25% of facilities (n = 3) offered VIA every day. Every district had at least one facility that offered cervical cytology and VIA. All facilities that offered VIA also offered cervical cytology screening (Fig 3A).

### Treatment Services

Every district had at least one facility that offers VIA screening immediately followed by cryotherapy (Fig 3B). Colposcopy is available in only three of six districts (Orange Walk, Belize, and Toledo; Fig 3C). Biopsies are available in every district except Corozal; this includes Cayo and Stann Creek, districts in which colposcopy was not available (Fig 3D). Loop electrosurgical excision procedure is available in every district except Corozal and Toledo; this includes Cayo and Stann Creek, districts in which colposcopy was not available (Fig 3E).

Regarding tertiary services, simple hysterectomy was available in every region at the regional hospitals but was not available in Corozal or Toledo, districts lacking a regional health care center (Fig 3F). Radical hysterectomy and chemotherapy were available only in Belize and Stann Creek districts (Figs 3G and 3I). Radiotherapy, the primary treatment modality for locally advanced cervical cancer, was not available in Belize. The national pathology facility in Belize district, located on the same campus as the national referral hospital, was the sole facility with pathology in-house (Fig 3H).

### Pathology Equipment and Supplies Availability

**FIG 4:**
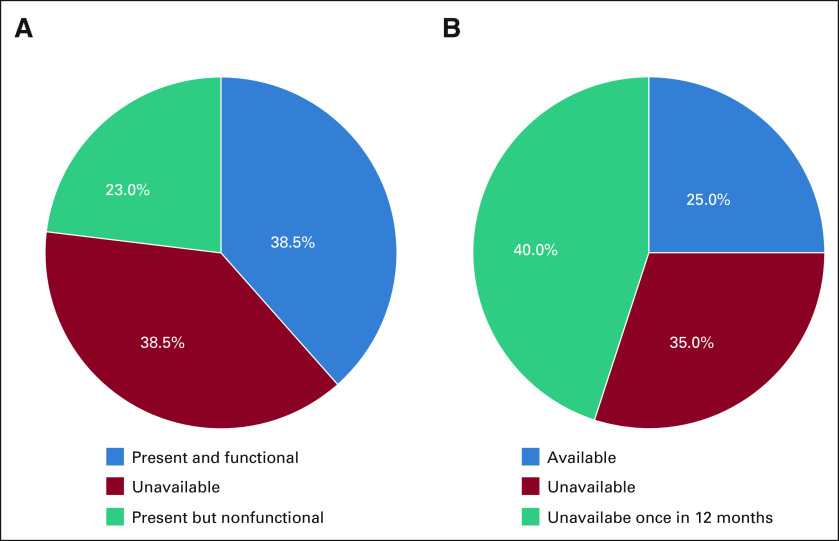
(A) Equipment status is determined by the presence and functional status of the surveyed equipment at the time of the assessment. (B) Supplies status was determined by the availability of surveyed supplies at the time of the assessment and over the past 12 months.

At the national pathology facility, 38.5% (n = 5) of essential equipment was present and functional at the time of assessment; 38.5% (n = 5) of equipment was not present and 23% (n = 3) was present but nonfunctional (Fig 4A). Thirty-five percent (n = 7) of supplies were available at the time of assessment, and 75% (n = 15) of supplies were unavailable at least once during the past year (Fig 4B).

## DISCUSSION

This study is the first to comprehensively describe the capacity of cervical cancer prevention and treatment infrastructure in the Belizean public health care system by providing estimates of human resources, screening capacity, service availability, and pathology infrastructure. These findings create a snapshot of the current state of cervical cancer management in Belize and can be used to set attainable goals for increasing cervical cancer prevention efforts, generate hypotheses for the reasons of cervical cancer burden, and develop a framework for capacity building in other nations.

The cervical cancer screening workforce in Belize relies primarily on nurses and general practitioners to perform the majority of cervical cytology and VIA procedures. Although a benchmark for screening workforce density has yet to be described, the present estimate of the Belizean screening workforce would require that each professional screens 359 women per year to achieve 100% screening coverage, a target which is not feasible in the current work conditions. Furthermore, a minority of the outreach workforce serves the rural regions where more than half of Belizeans reside.^13^ Potential solutions to the limited ability of the cervical cancer screening workforce to engage the rural population include employing community health workers to bolster the screening workforce. Some countries across Africa, Asia, and South and Central America have employed community health workers without formal training as health care providers to conduct cervical cancer screening.^14^ HPV self-sampling is another innovative screening approach that can be implemented in rural areas to further improve screening access without relying on a larger screening workforce. Self-sampling also enables increased compliance among women who would not otherwise present for screening and has the additional advantage of being conducted in the privacy of one's home.^15^ A study of women in rural Newfoundland found that cervical cancer screening rates increased significantly by 15.2% in those who were able to self-collect samples when compared with a control intervention (provision of educational materials).^16^ Similar observations were made in rural Cameroon, supporting the efficacy of self-collect samples in low- and middle-income countries as well.^17^

Although workforce density is a critical factor that affects timely access to care when needed, the frequency with which a particular service is offered provides additional context to the question of access. In Belize, 50% of the facilities that offer cervical cytology or VIA screening services do so only once per week. Approximately 42% of clinics offer cervical cytology daily, whereas only 25% of the clinics offered VIA and cryotherapy daily. Increasing screening coverage in Belize could be accomplished by increasing the proportion of facilities offering VIA daily. Without building new facilities or hiring more personnel, shifting priorities of health care workers toward offering screening more consistently could improve access. Prioritizing increasing the frequency of VIA screening could also address potential loss to follow-up and lack of access to early-stage treatment. VIA been shown to be more cost effective and easier to adopt than cervical cytology, and VIA results are available immediately, eliminating the risk of loss to follow-up by enabling same-day screening and treatment.^18^ Data from screening programs in Malawi and Honduras suggest that delays in screening and subsequent treatment result in a significant loss to follow-up rate.^19,20^

Belize has already taken great strides to make essential cervical cancer management services available in the public sector. Bychkovksy and colleagues found that Belize was among 22 other Latin American countries that offered cervical cytology services and among 15 other countries that offer VIA in the public sector (92% and 58% of countries analyzed, respectively).^21^ By 2018, Belize had 12 VIA programs in the public sector alone, with plans to scale up VIA availability to a total of 15 public and private centers by 2020, according to the Cancer Prevention and Control Strategic Plan.^21^ Although all of the six districts in Belize offer standard cervical cytology and VIA, the present analysis of treatment options has highlighted a decrease in geographically equitable availability as services become more advanced (Fig 3). The most advanced treatments options offered in Belize, including radical hysterectomy and chemotherapy, are only offered in the two centrally located districts, building an accessibility barrier between the remainder of the country's population and critical treatments. This geographic barrier also poses a socioeconomic challenge, as travel costs and costs of not working introduce another obstacle to accessing care. The unequal distribution of advanced treatment options may be one explanation for the persistent high morbidity and mortality rates of cervical cancer in Belize in comparison to other low-income and Latin American countries, despite widespread screen-and-treat services.^9,10^ This pattern is similar to that which has been documented over the past decade in Zambia, a low-income country in Africa that has been working to improve cervical cancer infrastructure. In Zambia, only one hospital offers radical hysterectomy and radiation therapy, and this hospital also acts as the primary chemotherapy center for the country.^11^ Within both Belize and Zambia, the lack of nationwide distribution of advanced treatment options restricts treatment options for those diagnosed with advanced cervical cancer. Although this assessment did not consider the role of surgical and radiation services offered to Belizean citizens in neighboring countries, international travel may be prohibitively expensive, further widening health care disparities. Future efforts to improve cervical cancer outcomes in Belize should involve implementing more advanced treatment options throughout Belize and developing in-country radiotherapy services, which is an arduous and costly endeavor. Radiotherapy access is generally limited in Caribbean countries—an analysis by the Pan American Health Organization in 2013 found that only four of 13 countries surveyed offered radiotherapy.^22^ Although Belize is years away from implementing radiotherapy services to treat cervical cancer, their cooperation with the International Atomic Energy Association and recent legislation supporting the safe introduction of nuclear technology is an important step forward in offering comprehensive cancer care.^23^ Although prevention is the most cost-effective and efficacious method to reduce cervical cancer mortality, the WHO recognizes that scaling treatment services through training more personnel or introducing new technologies plays an important role in addressing the burden of cervical cancer on low- and middle-income countries.^24^

Supporting the advancements made in screening and treatment services, the foundation of effective cervical cancer control and national cancer management is sustainable pathology infrastructure to process HPV samples, as well as cytology and histology services. In Belize, where the national pathology facility processes most cytology and tissue samples in the country, delays or challenges in the pathology program could drastically affect the entirety of the cervical cancer prevention and management system. In Figure 4, the equipment and supplies at the national pathology facility were summarized. More than half of critical equipment were unavailable or not functional at the time of assessment and 75% of supplies were unavailable at some point in the past year. These resource constraints are a bottleneck in the cervical cancer management strategy. Delaying communication of cytology and pathology results has been shown to cause unnecessary progression of disease.^25^ Since reliability of pathology resource availability in Belize is low and cytology accuracy is variable, there may be a stronger impetus for investing in decentralized screening such as VIA or other techniques that do not rely on pathology infrastructure, such as HPV-based strategies.^26-28^ Furthermore, the WHO has advocated for HPV-based testing or VIA in countries where cervical cytology programs are not sustainable.^29^

The main limitation of this investigation was the lack of sampling of rural health care centers. The health care centers sampled were primarily urban health care centers, as well as regional hospitals and the national referral hospital (Table 2). Although this sampling strategy provided a national perspective of the services offered in Belize, it did not adequately evaluate the screening workforce, outreach workforce, and screening policies at rural health care centers. Therefore, data included in this study lack information on cervical cancer screening and treatment delivery in rural locations in Belize outside of the measurement of health care workers that participate in outreach screening. Furthermore, because there was only one facility that met the inclusion criteria located in Toledo district, workforce and service availability estimates might not be well represented from this area. Also, many women receive preventative health care services in the private sector, ranging from cervical cytology and VIA to HPV DNA testing, although services in the private sector are prohibitively expensive for many Belizeans. There are also limitations in the HFA instrument. Although HFAs characterized the critical aspects of cervical management capacity, they may not adequately describe all aspects of capacity such as quality assurance practices and other important services (eg, frozen sectioning and immunohistochemistry). The absence of analysis of cultural and societal factors influencing obtaining screening and treatment for cervical cancer is another potential source of important information not reflected in the collected data. Although understanding cervical cancer prevention and treatment infrastructure is a key step in addressing the burden of disease in Belize, an assessment of public awareness of and attitudes toward cervical management is equally important.

In conclusion, over the past decade, Belize has implemented a number of cervical cancer prevention and treatment programs throughout all regions to address increasing burden of disease. This study elucidated that the extent of benefits from these programs is limited by workforce constraints at the local level and pathology infrastructure at the national level. Workforce growth and training and implementation of decentralized screening services may improve cervical cancer management and support sustainable services delivery at local clinics, regional treatment centers, and the national pathology facility. Belize has a more-than-adequate foundation of health care services that can be built upon through future developments. Future research incorporating a qualitative assessment of health care workers and administrators will allow for better contextualization of findings to ensure that future implementations of Belize bring us closer to a cervical cancer–free future.
